# An Inducible *Microbacterium* Prophage vB_MoxS-R1 Represents a Novel Lineage of Siphovirus

**DOI:** 10.3390/v14040731

**Published:** 2022-03-31

**Authors:** Hongrui Zheng, Binbin Liu, Yongle Xu, Zefeng Zhang, Hongcong Man, Jihua Liu, Feng Chen

**Affiliations:** 1 Institute of Marine Science and Technology, Shandong University, Qingdao 266000, China; zhenghr163@163.com (H.Z.); lbbkk187@163.com (B.L.); zfengbio@126.com (Z.Z.); 15898836092@163.com (H.M.); 2 Fujian Key Laboratory of Marine Carbon Sequestration, Xiamen University, Xiamen 361000, China; 3 Southern Marine Science and Engineering Guangdong Laboratory (Zhuhai), Zhuhai 519000, China; 4 Joint Laboratory for Ocean Research and Education at Dalhousie University, Shandong University and Xiamen University, Qingdao 266237, China; 5 Institute of Marine and Environmental Technology, University of Maryland Center for Environmental Science, Baltimore, MD 21202, USA; chenf@umces.edu

**Keywords:** *Microbacterium* prophage, comparative genomic analysis, new phage genus, transcriptional regulator, endonuclease

## Abstract

Lytic and lysogenic infections are the main strategies used by viruses to interact with microbial hosts. The genetic information of prophages provides insights into the nature of phages and their potential influences on hosts. Here, the siphovirus vB_MoxS-R1 was induced from a *Microbacterium* strain isolated from an estuarine *Synechococcus* culture. vB_MoxS-R1 has a high replication capability, with an estimated burst size of 2000 virions per cell. vB_MoxS-R1 represents a novel phage genus-based genomic analysis. Six transcriptional regulator (TR) genes were predicted in the vB_MoxS-R1 genome. Four of these TR genes are involved in stress responses, virulence and amino acid transportation in bacteria, suggesting that they may play roles in regulating the host cell metabolism in response to external environmental changes. A glycerophosphodiester phosphodiesterase gene related to phosphorus acquisition was also identified in the vB_MoxS-R1 genome. The presence of six TR genes and the phosphorus-acquisition gene suggests that prophage vB_MoxS-R1 has the potential to influence survival and adaptation of its host during lysogeny. Possession of four endonuclease genes in the prophage genome suggests that vB_MoxS-R1 is likely involved in DNA recombination or gene conversion and further influences host evolution.

## 1. Introduction

Viruses are the most abundant biological entities in the oceans [1,2,3], and they play important roles in influencing the community composition, biomass and genetic diversity of their hosts [3,4,5,6]. Metagenomic studies have shown that marine viruses have vast uncharacterized levels of genetic diversity [7,8]. The lack of viral reference genomes in databases limits the study of unknown viral sequences. The isolation and genomic analyses of new viruses are of great importance for further understanding the genetic diversity and biological features of unknown viruses [9,10].

Most viral characterizations have focused on lytic viruses. However, there are also many viruses hiding in hosts, integrating their genomic DNA into the host chromosomes as prophages and playing roles in bacterial life cycles [11]. Prophages can alter the biological characteristics of their hosts, thereby eliciting a variety of effects on the host and its surroundings [12,13,14,15,16]. Prophages are also viewed as “dangerous molecular time bombs” in lysogenic bacteria [17,18]. Under certain circumstances, prophages may be induced to lyse the host cells and become free phage particles. The occurrence of lysogeny is a controversial topic. In previous studies, lysogeny was thought to be a survival strategy for bacteria and phages under low-energy source conditions [17]. Conversely, the new Piggyback-the-Winner model reveals that lysogeny predominates at high bacterial densities [19,20]. Lysogeny is a common phenomenon that occurs in almost all habitats [14,21]. Approximately 47% of isolated terrestrial bacteria are lysogenic, and 28–71% of marine bacterial isolates contain prophages [22,23,24,25,26]. Prophages can protect the host from lytic infection by bacteriophages of the same type, and they are involved in intermediate horizontal gene transfer within hosts, thereby affecting the evolution, diversity and biological characteristics of the bacterial community [17,27,28].

Actinobacteria is a diverse and important group of microorganisms in both aquatic and terrestrial ecosystems [29,30]. Actinobacteria contain a full set of enzymes that can degrade refractory polymer substances, such as cellulose and chitin, thereby playing important roles in carbon cycling [31,32,33,34,35,36,37]. As ubiquitous symbionts of eukaryotes [38,39,40,41], Actinobacteria serve as defensive mutualists or aid hosts with nutrient acquisition. Moreover, members of Actinobacteria are associated with cyanobacteria. In studies on photoautotroph–heterotroph interactions, Actinobacteria usually represent one of the dominant bacterial taxa in cyanobacterial cultures [42,43]. Furthermore, Actinobacteria accounts for 20% of the bacterial community and exhibit specific associations with increased cyanobacterial abundance in estuarine ecosystems [44]. The genus *Microbacterium*, belonging to the family *Microbacteriaceae* of Actinobacteria, is a very heterogeneous group and includes more than 55 species, most of which are soil organisms that specialize in degrading complex organic substrates [45,46,47]. *Microbacterium* members are also important constituents in marine habitats, such as seawater, deep-sea sediments and marine sponges [48,49,50].

A large number of *Microbacterium* phages have been isolated from various environments [51]. At the time of writing, 341 *Microbacterium* phages isolated using seven different *Microbacterium* hosts have been sequenced, and they are divided into 19 clusters (https://phagesdb.org/ (accessed on 17 July 2020)) [52]. Among the 341 sequenced *Microbacterium* phages, only four are temperate, with three (Floof, Percival and Zeta1847) grouped into the EH cluster and one (Min1) being a singleton [51,53]. Min1 was the first reported *Microbacterium* phage, and it has a temperate life cycle in which it integrates into a stable plasmid, called pMN1, in *Microbacterium nematophilum* CBX102 [53]. More detailed genomic analyses are needed to understand the genetic diversity of temperate *Microbacterium* phages and *Microbacterium*–phage interactions in different ecosystems.

Here, we report a novel inducible prophage vB_MoxS-R1 from *Microbacterium* strain R1 that was isolated from an estuarine *Synechococcus* culture. It has a large burst size and a narrow host range. Network and phylogenetic analysis showed that vB_MoxS-R1 represents a novel lineage of siphovirus. A genome composition analysis revealed that vB_MoxS-R1 may have the potential to regulate metabolism and influence the evolution of the host.

## 2. Materials and Methods

### 2.1. Host Bacterial Cultivation and Prophage Induction

*Microbacterium oxydans* R1 (GenBank accession no. NZ_JADDUD000000000.1) was isolated from the *Synechococcus* sp. CBW1107 [54,55] culture using RO medium as described by Yurkov et al. [56]. The exponentially growing culture (OD_600_ = 0.2) of *Microbacterium oxydans* R1 was split equally into six flasks, with each containing 100 mL culture, three of which were treated with mitomycin C at a final concentration of 0.5 mg/L, with the other three were used as controls. After treatment with mitomycin C for half an hour, bacterial cells in treatment and control groups were both washed twice by centrifugation at 6000× *g* for 10 min and resuspended in fresh RO medium. Both groups were incubated continually at 28 ℃ in a shaker with a speed of 160 rpm. Subsamples of bacterial cells and phage particles were taken every 2 h for the first 12 h, every 4 h for 12–24 h and every 8 h for 24–48 h. Bacterial samples were fixed with glutaraldehyde at a final concentration of 0.5% for 15 min in the dark. Phage samples were filtered through 0.45 μm filters (Millipore, Bedford, MA, USA) and fixed in darkness with glutaraldehyde at a final concentration of 0.5% for 15 min. All subsamples were stained by SYBR Green I (Molecular Probes-Invitrogen, OR, USA). Bacterial concentrations were analyzed by a BD AccuriTM C6 Plus Flow cytometer (Becton Dickinson, San Jose, CA, USA), and phage concentrations were analyzed by CytoFLEX (Beckman Coulter, Krefeld, Germany).

### 2.2. Phage Amplification and Purification

For prophage amplification, *Microbacterium oxydans* R1 was incubated in 1 L of RO medium at 28 °C in a shaker at 160 rpm. Mitomycin C was added at a final concentration of 0.5 mg/L when the bacterial culture reached the exponential growth phase (OD_600_ = 0.4). At 30 min after the mitomycin C treatment, bacterial cells were washed twice by centrifugation at 10,000× *g* for 10 min and resuspended in 1 L of fresh RO medium. Phage lysates were harvested and purified as described by Xu et al. [57], with modifications. Briefly, phage lysates were treated with both RNase A and DNase I at final concentrations of 2 μg/mL at room temperature for 1 h. Afterward, the NaCl concentration was adjusted to 1 M, and the lysate was placed in an ice bath for 0.5 h. The phage lysate was then centrifuged at 10,000× *g* for 20 min. Supernatants were collected and filtered through 0.45 μm filters (Millipore, Bedford, MA, USA) to remove host cells and cellular debris. The filtrate was treated with PEG8000 at a final concentration of 100 g/L and incubated overnight at 4 °C. Phage particles were precipitated by centrifugation at 12,000× *g* for 1 h and resuspended with 2 mL of TM buffer (20 mM Tris-Cl and 10 mM MgSO_4_). Phage particles were then purified by ultracentrifugation in CsCl density gradients at 200,000× *g* for 24 h in a SW 41Ti rotor (Beckman Optima L-100XP, Beckman Coulter, CA, USA). Visible viral bands were extracted and desalted using a 30-kDa cut-off in a centrifugal ultrafiltration unit (Millipore, Bedford, MA, USA). Purified phage suspensions were stored at 4 °C.

### 2.3. Transmission Electron Microscopy (TEM) Observation

A drop of purified phage suspension was adsorbed on carbon-coated copper grids for 20 min and then stained with 1% phosphotungstic acid for 1 min. Each sample was dried for 2 h and examined using a Tecnai G2 Spirit BioTwin transmission electron microscope (FEI Thermo Fisher Scientific, Eindhoven, The Netherlands).

### 2.4. Host Range Determination

The cross infectivity of vB_MoxS-R1 was tested against 13 bacterial strains, which were also isolated from estuarine *Synechococcus* cultures [55], belonging to *Microbacterium*, *Arenibacter*, *Halomonas*, *Marinobacter*, *Mesorhizobium*, *Muricauda*, *Nitratireductor* and *Sphingomonas* (Table 1). The spot assay method was used to determine the cross infectivity of vB_MoxS-R1. Briefly, 1 mL of exponentially growing bacterial culture was mixed with 5 mL of molten RO medium with 0.5% agar and poured onto solid RO medium with 1.5% agar in a Petri dish. After the bacterial cultures were incubated overnight at 28 °C, 5 μL of purified phage suspension was spotted onto the bacterial lawn in triplicate. All spotted plates were incubated at 28 °C and monitored after 24 h and 48 h. Phage infection was indicated based on the bacterial lysis in the lawn. If no plaques were observed in the lawn, further investigation was carried out to explore whether phage vB_MoxS-R1 was lysogenic in the tested bacterial strains. Briefly, bacterial strains were incubated in RO medium at 28 °C in a shaker at 160 rpm. Exponentially growing culture (OD600 = 0.4) of each tested strain was split equally into six flasks, three of which were inoculated with phage suspensions at a multiplicity of infection (MOI) of 1 and assigned as the treated group; the other three were used as the control group. After putative phage adsorption for 0.5 h, both groups were washed twice by centrifugation at 6000× *g* for 10 min and resuspended in fresh RO medium. All flasks were incubated at 28 °C in a shaker at 160 rpm, and bacterial cell samples were taken at 0.5, 2 and 4 h. The entry and replication of vB_MoxS-R1 in the bacterial cells was detected by the presence and copy variation of the major capsid protein gene (*mcp*) of vB_MoxS-R1. Primers targeting the *mcp* of vB_MoxS-R1 were designed using Primer Premier 5. The primers R1-F (5′-ATCGTCCTCCCGAACCTG-3′) and R1-R (5′-GCGTGTCGCTGTCGTAGTC-3′) were designed for *mcp* presence detection by PCR, whereas primers R1qpcr-F (5′-GACGCCTGTCCAGTTTCA-3′) and R1qpcr-R (5′-GCGTGTCGCTGTCGTAG-3′) were designed for *mcp* copy quantification by SYBR Green-based quantitative real-time PCR (ABclonal, Wuhan, China) [58,59]. Phage presence within the cells was first detected by PCR amplification of the vB_MoxS-R1 *mcp.* Once positive, the *mcp* copy number was further quantified to determine whether vB_MoxS-R1 adsorbed into or replicated in the cells.

### 2.5. Phage DNA Extraction and Genome Sequencing

Purified phage particles were treated with proteinase K (150 μg/mL), EDTA (5 mM, pH 8.0) and sodium dodecyl sulfate (1% *w*/*v*) and then incubated at 55 °C for 3 h. The phage DNA was extracted using the phenol/chloroform method [10,18], precipitated with ethanol, dissolved in sterile Milli-Q water and stored at −20 °C. The genomic DNA was then sequenced by Shanghai Majorbio Bio-pharm Technology Co., Ltd. Raw reads were quality-checked using FastQC and trimmed using the FASTX-Toolkit. The clean reads were assembled using an IDBA-UD algorithm [60]. The phage genome sequence was submitted to the GenBank database under accession no. MW073100.

### 2.6. Genome Annotation

Open reading frames (ORFs) of the prophage genome were predicted using the GeneMarkS online server (http://exon.gatech.edu/GeneMark/ (accessed on 12 March 2020)), RAST (http://rast.nmpdr.org/ (accessed on 12 March 2020)) and Meta Gene Annotator (http://metagene.nig.ac.jp/ (accessed on 12 March 2020)). The ORF homologs of the induced phage in other microorganisms were obtained using a BLASTP search against the NCBI non-redundant (NR) database at a cut-off e-value of <10^−3^ [61]. A conserved domain search against the NCBI Conserved Domain Database was conducted to assist the annotation of each ORF [62]. The tRNA sequences were identified using tRNAscan-SE (http://lowelab.ucsc.edu/tRNAscan-SE (accessed on 15 March 2020)) [63,64].

### 2.7. Phage Genome Network Analyses and Phylogenetic Analyses

A total of 11,460 viral genomes (577,786 proteins) were downloaded from NCBI Viral RefSeq, and 341 *Microbacterium* phage genomes (14,337 proteins) were downloaded from the Actinobacteriophage Database (https://phagesdb.org/ (accessed on 17 July 2020)) [52]. Proteins were compared using all-versus-all BLASTP with a threshold e-value of 10^−5^ and a bit score of >50, after which protein clusters were defined by using the Markov clustering algorithm in both viral genome assemblages as previously described [65,66]. vConTACT 2.0 was used to calculate the similarity score between each pair of viral genomes, and ClusterONE was used to identify the viral cluster [67,68]. The two networks revealing the relation between the prophage genome and those 11,460 viral genomes and the *Microbacterium* phage genomes were visualized using Cytoscape 3.8.2 [69,70]. Network relation between genomes of vB_MoxS-R1 and its related phages was further characterized in levels of the genomic nucleotide similarity using VIRIDIC (http://rhea.icbm.uni-oldenburg.de/VIRIDIC/ (accessed on 14 January 2022)) [71] and ORF homology using NCBI BLASTP. Phylogenetic analyses of the bacterial host and phages were conducted on the basis of the 16S rRNA gene, phage genome, major capsid protein and terminase large-subunit sequences. The ORF homology between vB_MoxS-R1 and the prophage-like fragment of *Microbacterium* sp. UCD-TDU was identified using BLASTP (version 2.4.0+) with a threshold e-value of 10^−3^, a bit score of >40 and a minimum amino acid length of 30, as previously described [72,73]. The Virus Classification and Tree Building Online Resource (VICTOR, https://ggdc.dsmz.de/victor.php (accessed on 1 January 2022)) and Mega7.0 software package were used for phylogenetic analyses [74,75,76]. Phylogenomic analyses were conducted using the Genome-BLAST distance phylogeny (GBDP) method [76,77]. The maximum likelihood and neighbor-joining methods were used to construct the 16S rRNA gene, major capsid protein and terminase large subunit phylogenetic trees with 1000 bootstrap replicates.

## 3. Results and Discussion

### 3.1. Induction and Basic Biological Features of Phage vB_MoxS-R1

**Figure 1:**
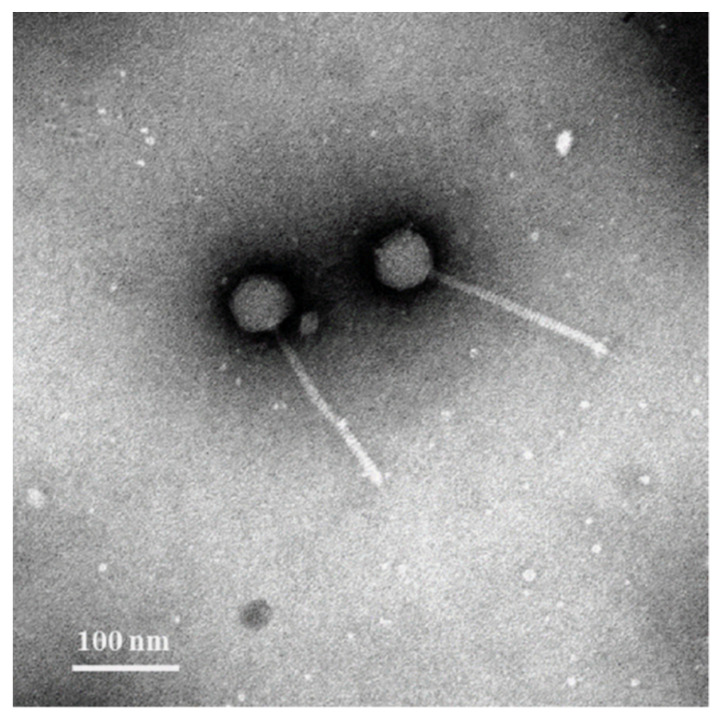
Transmission electron microscopy image of vB_MoxS-R1.

**Figure 2:**
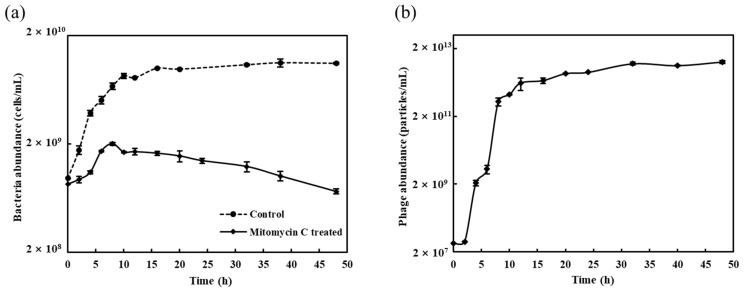
vB_MoxS-R1 induction from *Microbacterium oxydans* R1 by mitomycin C. (**a**) Effect of mitomycin C treatment on growth of *Microbacterium oxydans* R1. (**b**) Viral particle yields following mitomycin C induction of *Microbacterium oxydans* R1.

vB_MoxS-R1 was induced in *Microbacterium oxydans* R1, which was isolated from a *Synechococcus* sp. CBW1107 culture [55]. TEM observations revealed that vB_MoxS-R1 is a siphovirus with an isometric icosahedral head (approximately 61 nm in diameter) and a long, flexible tail (approximately 178 nm in length and 11 nm in width) (Figure 1). After treatment with mitomycin C, the growth of *Microbacterium oxydans* R1 was significantly inhibited (Figure 2a). Phage particles begin to release after 2 h and then almost achieved the maximum at 12 h. During this period, the abundance of the phage particles increased by five orders of magnitude (Figure 2b). If all viruses were produced by the lysis of mitomycin C-treated *Microbacterium oxydans* R1 cells at the beginning of incubation, ignoring the probable fraction of released phage particles becoming relysogenic into bacterial cells, the burst size of vB_MoxS-R1 was at least 2000 virions per cell, which is 5–400 times larger than those of other mitomycin C-induced prophages (5–435 virions per cell) [11,78,79,80,81]. The large burst size of vB_MoxS-R1 reveals its high replication capability. A cross-infectivity test with 13 *Synechococcus*-associated bacterial strains showed that no plaques were observed on the host lawn after adding phage suspensions, which indicates that vB_MoxS-R1 did not lyse those bacterial strains (Table 1). We further investigated whether vB_MoxS-R1 was lysogenic into the tested bacterial strains by determining the presence and copy number of the vB_MoxS-R1 *mcp* in the bacterial cells after phage adsorption. The vB_MoxS-R1 *mcp* was only tested positive in phage-treated and control samples of *Microbacterium oxydans* R1, CBW1101-8 and CBW1101-9, whereas the other 10 strains were proved to be not infected by vB_MoxS-R1. The *mcp* sequences amplified from the control samples of CBW1101-8 and CBW1101-9 were 100% identical with that of vB_MoxS-R1, indicating that the same or at least similar prophages were lysogenic in bacterial cells of CBW1101-8 and CBW1101-9. The *mcp* copy number was further quantified to determine whether vB_MoxS-R1 adsorbed into or replicated in the cells of *Microbacterium oxydans* R1, CBW1101-8 and CBW1101-9. Increased *mcp* copies were observed in all phage-treated cells of the three *Microbacterium* strains at all sampling times (0.5, 2 and 4 h after phage adsorption). The *mcp* copies in the phage-treated samples increased by 7–50% compared to the control samples, indicating that vB_MoxS-R1 entered the *Microbacterium oxydans* bacterial cells but maintained low levels (Table S1). Based on the above, we speculated that vB_MoxS-R1 was lysogenic in *Microbacterium oxydans* R1, CBW1101-8 and CBW1101-9 during the infection (Table 1).

### 3.2. Genomic Properties and Structure of Phage vB_MoxS-R1

The genome of vB_MoxS-R1 is 42.56 kb with a 63.7% G + C content, whereas the bacteria *Microbacterium oxydans* R1 genome is 3.49 Mb with a 68.2% G + C content. The vB_MoxS-R1 genome accounts for 1% of the host genome and has a slightly lower G + C content than its host. Among the 341 sequenced *Microbacterium* phages, the genome size and G + C content of vB_MoxS-R1 fall into the most common ranges of the sequenced *Microbacterium* phages (https://phagesdb.org/ (accessed on 17 July 2020)). A total of 77 ORFs and two tRNA genes were predicted in the vB_MoxS-R1 genome (Table S2). In total, 63 ORFs use ATG as the start codon, whereas 13 ORFs and one ORF start with GTG and TTG codons, respectively. Additionally, 67 ORFs have homologs in the NCBI NR database. Among them, only 10 ORFs show homology to genes predicted in phage genomes, with 6 being from *Microbacterium* phages. In addition, a BLASTn search of the vB_MoxS-R1 complete genome sequence against the NCBI nucleotide collection (NR/NT) database revealed that vB_MoxS-R1 has no significant similarity to phages. The ORF and genome sequence homology-based identifications both indicated that vB_MoxS-R1 represents a novel siphovirus lineage.

**Figure 3:**
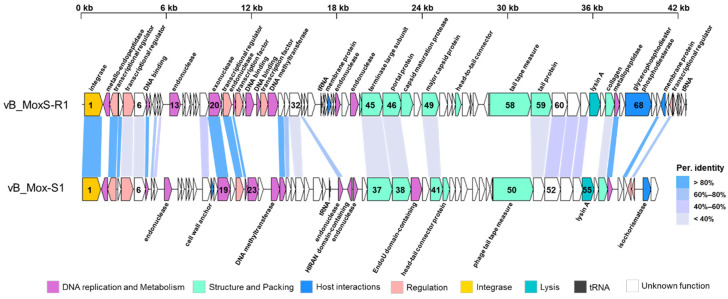
Genome organization and comparison of vB_MoxS-R1 and vB_Mox-S1. The direction of open reading frame (ORF) transcription is depicted by a leftward- or rightward-oriented arrow.

Despite having few similarities with phages, a large number of vB_MoxS-R1 ORFs (62, 80.52%) showed homology with genes predicted in *Microbacterium* spp. genomes (Table S2). Among those *Microbacterium* spp., vB_MoxS-R1 shared the maximum number of ORF homologs with *Microbacterium* sp. UCD-TDU. By comparing genome sequences between vB_MoxS-R1 and *Microbacterium* sp. UCD-TDU using Circoletto (http://tools.bat.infspire.org/circoletto/ (accessed on 18 March 2020)), a prophage-like region of 40.55 kb was identified in the *Microbacterium* sp. UCD-TDU genome (Figure S1), hereafter termed vB_Mox-S1 in this study. A total of 24 ORFs in the genome of vB_MoxS-R1 are homologous to those of vB_Mox-S1 (Figure 3). Moreover, the G + C content of vB_Mox-S1 is 63.7%, which is similar to that of vB_MoxS-R1.

The 31 ORFs with predictable functions in the vB_MoxS-R1 genome were divided into the five following categories: genes related to DNA replication and metabolism (11 ORFs), structure formation (nine ORFs), regulation and modification of host metabolism (nine ORFs), integrase (one ORF) and lysis (one ORF) (Figure 3, Table S2). Among the 24 vB_MoxS-R1 ORFs that showed homology with those of vB_Mox-S1, four are related to DNA replication and metabolism, four are involved in structural formation and packing, four are regulatory genes, one is related to integration and 11 have unknown functions. The amino acid sequence identities shared between the vB_MoxS-R1 and vB_Mox-S1 ORF homologs related to DNA replication and metabolism, integrase and regulation (30.08% to 98.77%) are much greater than those of homologs related to lysis, structural formation and packing (26.44% to 35.78%), indicating different evolutionary paths for genes in different categories.

### 3.3. vB_MoxS-R1 Represents a New Phage Genus

**Figure 4:**
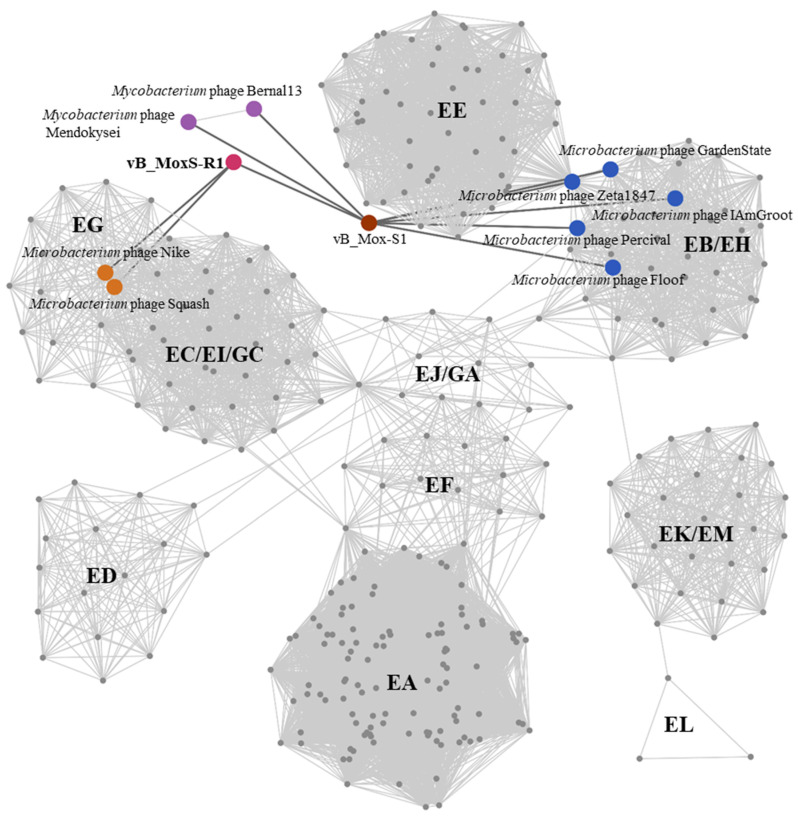
Protein-sharing viral network of vB_MoxS-R1, vB_Mox-S1 and 341 *Microbacterium* phages, as well as two vB_Mox-S1-related *Mycobacterium* phages with a pairing-similarity score >1. Each node represents the genome of a phage. Edges represent the similarity scores of shared proteins between phages, and edges related to vB_MoxS-R1 and vB_Mox-S1 are displayed in bold and colored in dark gray. The nodes of vB_MoxS-R1, vB_Mox-S1 and their related phages are enlarged in different colors according to their phylotypes. Group names of *Microbacterium* phages are shown on each cluster.

**Figure 5:**
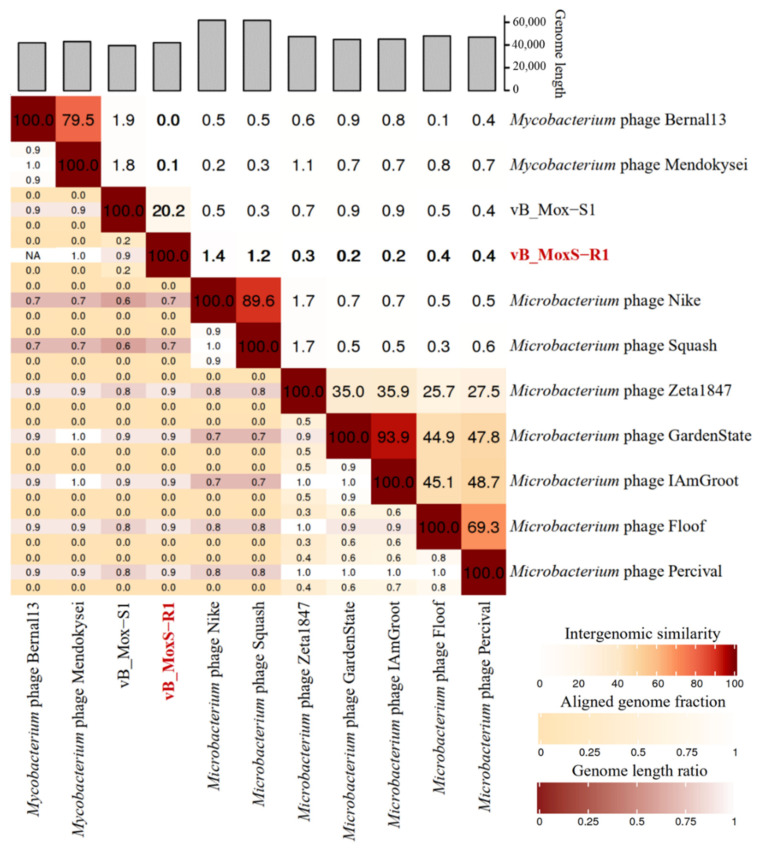
Intergenomic similarity between vB_MoxS-R1 and vB_Mox-S1 and their network-related phages calculated using VIRIDIC. The similarities of vB_MoxS-R1 with other phages are displayed in bold. The right half of this heatmap represents the similarity values between genomes. The left half of this heatmap represents the aligned genome fraction and genome length ratio.

Viral genome network analysis between vB_MoxS-R1 and those in the Viral RefSeq database (Figure S2) showed that vB_MoxS-R1 was only related to vB_Mox-S1. Considering that the Viral RefSeq database only contains a limited number of *Microbacterium* phages, further genomic network analysis was performed among vB_MoxS-R1, vB_Mox-S1 and 341 *Microbacterium* phages retrieved from the Actinobacteriophage Database, as well as two *Mycobacterium* phages related to vB_Mox-S1 (Figure S2). vB_MoxS-R1 was found to only be related to vB_Mox-S1 and two *Microbacterium* phages in the EG cluster, whereas vB_Mox-S1 was related to another five *Microbacterium* phages in the EH cluster and the two *Mycobacterium* phages (Figure 4). Comparative analysis of genomic nucleotide similarity and ORF homology of vB_MoxS-R1 with vB_Mox-S1, two related *Microbacterium* phages, five EH cluster *Microbacterium* phages and two *Mycobacterium* phages that are related to vB_Mox-S1, showed that the genome sequence similarities between vB_MoxS-R1 and these phages were 0–20.2% (Figure 5), and ORF homologs take up 2.6–31.2% of the total vB_MoxS-R1 ORFs (Table 2). Except for vB_Mox-S1, only two to four ORFs in the other nine phage genomes showed homology with those of vB_MoxS-R1. The ORF amino acid identities between vB_MoxS-R1 and its two related *Microbacterium* phages (33.6–54.9%) were generally higher than those between vB_MoxS-R1- and vB_Mox-S1-related phages (24.9–43.4%) (Table 2). According to the recognized virus classification standards, viruses in the same genus should share >50% similarity in nucleotide sequence or > 40% ORF homologs [82,83], so we proposed that vB_MoxS-R1 is a new bacteriophage genus. The new genus is named *Syrbvirus* and has been submitted to ICTV.

**Figure 6:**
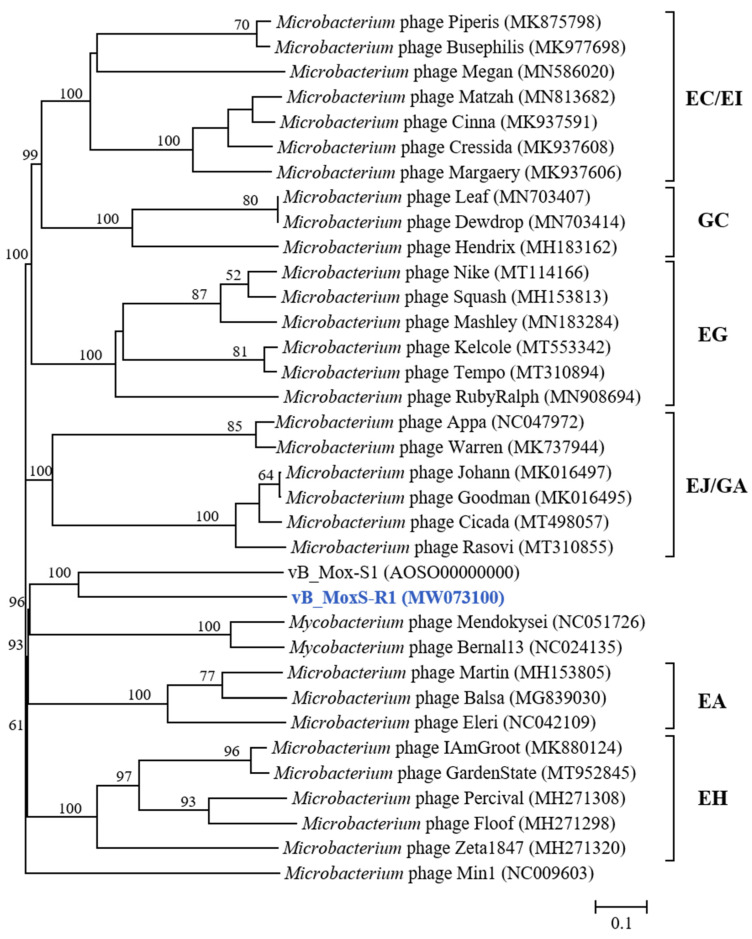
Phylogenomic tree of the *Microbacterium* phages and two vB_Mox-S1-related *Mycobacterium* phages. This tree was generated using the Genome-BLAST distance phylogeny (GBDP) method, and the number near each node is the GBDP pseudo-bootstrap support value from 100 replications (only values >50% are shown).

**Figure 7:**
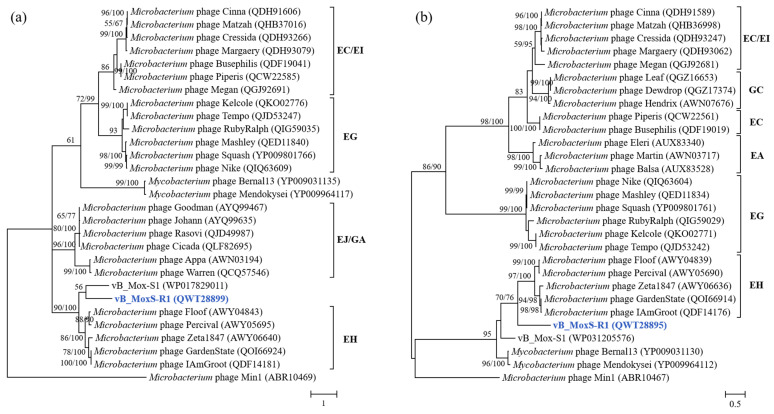
Unrooted maximum-likelihood phylogenetic trees of the major capsid protein (**a**) and terminase large subunit (**b**) of vB_MoxS-R1, *Microbacterium* phages and two *Mycobacterium* phages based on the amino acid sequences. The bootstrap values (maximum-likelihood/neighbor-joining) are shown near each node. Number of bootstrap replicates = 1000.

The phylogenomic analysis of vB_MoxS-R1, vB_Mox-S1, *Microbacterium* phages and the two vB_Mox-S1-related *Mycobacterium* phages revealed that vB_MoxS-R1 and vB_Mox-S1 both formed individual deep branches and represent new phage clusters (Figure 6). In addition, phylogenetic relationships with aspect to the major capsid and terminase large-subunit genes among vB_MoxS-R1, vB_Mox-S1, *Microbacterium* phages and the two vB_Mox-S1-related *Mycobacterium* phages were also assessed (Figure 7, Figure S3). Phylogenetic analyses of both genes (Figure 7) revealed that vB_MoxS-R1 and vB_Mox-S1 clustered together with the EH cluster but formed two deep branches. It is noteworthy that three of the five strains of the EH cluster are inducible prophages. The major capsid and terminase large-subunit genes are among the limited number of ORF homologs between the vB_MoxS-R1 and the EH cluster phages at low-amino-acid identities (21.8–32.7%) (Table 2). The limited number of distant homologs suggests horizontal gene transfer among those prophages during evolution. Phylogenetic analyses of the *Microbacterium* hosts of vB_MoxS-R1, the EH-cluster phages, the temperate phage Min 1 and *Microbacterium* sp. UCD-TDU using the 16S rRNA sequences revealed that they formed a cluster and were closely related (Figure S4), indicating that the prophage occurrence frequency may be related to the host taxa in the *Microbacterium* genus. Furthermore, *Microbacterium oxydans* R1 is more closely related to *Microbacterium* sp. UCD-TDU and the hosts of the EH phages than to the Min 1 host (Figure S4), which is in line with the phylogenetic analyses of the phage genes (Figure 6 and Figure 7).

### 3.4. DNA Repair and Modification

A total of 11 ORFs in the vB_MoxS-R1 genome are predicted to be involved in DNA metabolism (Figure 3, Table S2). The vB_MoxS-R1 genome lacks recognizable DNA polymerase, primase or helicase genes commonly found in lytic phages but encodes a set of genes related to DNA repair and modification. ORF2 encodes an IrrE/ImmA family metalloendopeptidase. As a crucial DNA repair regulatory protein, IrrE recognizes a broad range of DNA damage and acts as a “general switch” for DNA repair and protective pathways by regulating the expression of *recA* and *pprA*. The enhanced expression of *recA* and *pprA* stimulated by IrrE in response to ionizing radiation and UV light has been observed previously in *Deinococcus radiodurans* [84,85,86,87]. Moreover, RecA plays an important role in regulating the cellular SOS response and catalyzing homologous recombinational DNA repair in response to DNA damage [88,89,90]. In addition, IrrE contains the catalytic domain of the ImmA. Metallopeptidase ImmA can cleave the immunity repressor and then activate the conjugative transposon to regulate *recA* expression [91,92]. The DNA double-strand break response protein PprA plays a critical role in non-homologous end joining for DNA repair and in protecting against oxidative stress caused by UV radiation [85,88,93,94].

ORF29 was predicted to encode a protein that belongs to a family of cytosine-specific methyltransferases. DNA methylation is an important DNA modification [95]. DNA methyltransferases play vital roles in many cellular processes, such as DNA replication, protection, post-replicative mismatch repair, protein–DNA interaction and cell cycle [96,97,98]. Most prokaryotic DNA methyltransferases belong to the restriction–modification system [97]. Methyltransferases can protect specific DNA sequences from digestion by restriction endonucleases [97,99]. In addition, cytosine-specific DNA methylase plays a crucial role in viral maturation in infected *Escherichia coli* cells [100].

Four ORFs in the vB_MoxS-R1 genome, ORF13, ORF23, ORF41 and ORF43, encode proteins that are homologous to HNH endonucleases. The HNH endonucleases are common in bacteria and phages [101], and they include restriction, homing and structure-specific endonucleases, as well as DNA repair-associated enzymes [101,102]. In phage genomes, the HNH endonuclease gene is usually located close to a terminase gene [101,103]. The juxtaposition is highly conserved, and it may promote the occurrence of homologous recombination and gene conversion processes [101,103]. In the vB_MoxS-R1 genome, ORF41 and ORF43 are located close to terminase genes, whereas ORF13 and ORF23 are far away from the terminases. The presence of numerous endonuclease genes in the vB_MoxS-R1 genome suggests that vB_MoxS-R1 may play important roles in DNA recombination or gene conversion and in driving host evolution. Moreover, endonucleases and methyltransferases constitute restriction–modification systems in microorganisms [100]. In temperate phages, these systems protect bacterial cells from lysis by limiting prophage proliferation [104].

### 3.5. Diverse Transcriptional Regulators

Six ORFs (ORF3, ORF5, ORF22, ORF24, ORF28 and ORF73) in the vB_MoxS-R1 genome were predicted to encode different types of transcriptional regulators (TRs) (Figure 3, Table 3, Table S2). ORF5 encodes an ArsR-family regulatory protein, which is a substrate-responsive repressor of the arsenical resistance (Ars) operon’s transcription [105,106,107,108,109,110,111]. Arsenic is a toxic metalloid that is widely distributed in soil, freshwater, groundwater and seawater [106]. Many organisms, including bacteria, fungi, algae and plants, have developed arsenic-resistance mechanisms to survive arsenic-containing environments [112]. The expression of the phage ArsR may regulate arsenic’s effects on the host. ORF22 and ORF73 encode TRs containing HTH domains. As an ancient protein group, HTH proteins regulate the transcription of many biological processes, such as cell proliferation, DNA movement and circadian rhythm maintenance [113]. Members of the XRE TRs are involved in stress response and virulence [114,115,116]. ORF24 is predicted to be an ASCH domain-containing transcription factor. The ASCH domain functions as an RNA-binding domain during transcription, coactivation and RNA processing [117,118]. The ASCH domains of prokaryotes and phages are usually adjacent to the TRs’ HTH domains [118], and this occurs in the genome of vB_MoxS-R1. ORF3 and ORF28 encode TRs that remain to be identified. The presence of various TRs in the vB_MoxS-R1 genome suggests that vB_MoxS-R1 may regulate host metabolism in response to external environmental changes and enhance host adaptation. Three TRs in the vB_MoxS-R1 genome are associated with adversity resistance, and this may be attributed to the presence of corresponding stresses in the host surroundings. The expression of viral TRs would confer advantages for host survival under harsh environmental conditions. Moreover, the number of regulatory factors in the vB_MoxS-R1 genome is much greater than the average number of two present in prophages [17], and this indicates that vB_MoxS-R1 plays a greater role in regulating host metabolism than most prophages. Undefined TRs reflect the diversity levels of viral genes and the diverse regulatory roles of the viruses in host metabolism. 

### 3.6. Auxiliary Metabolic Genes

Phosphate is usually a nutrient-limiting factor for primary production in aquatic ecosystems [119,120]. In response to phosphorus-limited conditions, organisms evolved a series of complex strategies to improve their abilities to obtain and use phosphorus [119]. ORF68 in the vB_MoxS-R1 genome encodes a glycerophosphodiester phosphodiesterase that is related to phosphorus acquisition. Phage glycerophosphodiester phosphodiesterases are thought to participate in the host’s glycerol metabolism pathway and release phosphorus during phage infection, alleviating the host’s survival stress under phosphorus-limited conditions [119].

ORF39 and ORF71 were predicted to encode membrane proteins. Membrane proteins are important components of the bacterial cell wall. The expression of phage membrane proteins may modify the surface composition of the host and affect the host cell’s interactions with other phages, bacteria or predators in the surroundings [121,122].

### 3.7. Putative Frameshift in the Terminase Large Subunit

ORF44 and ORF45 were both predicted to encode part of the terminase large subunit. This redundancy may be caused by a frameshift that would produce two overlapping proteins in an appropriate ratio [123]. Frameshifts use the least genetic information to obtain several different proteins, which are not errors but common extensions of the genetic code [124]. According to the sequence alignment of ORF44 and ORF45 with the terminase large-subunit gene (*terL*) homologs (Figure S5a), it is speculated that the vB_MoxS-R1 *terL* uses a −1 frameshift, which is the best studied of the recoding events [123,124,125,126]. We speculate that the slippery sequence GGT-AGC obtains the nucleobase T and becomes GGT-TAG, and TAG is a stop codon that can terminate the translation process (Figure S5a). In long-tailed dsDNA phages, frameshifts in tail assembly genes are common [125,127], whereas frameshift mutations in *terL* are less reported. A *terL* segregated into two ORFs is once observed in the genome of *Gordonia* phage Nyceirae (GenBank no. KX557282.1); whether it is caused by frameshift remains to be studied. The putative frameshift in the vB_MoxS-R1 *terL* is only predicted according to the alignment of ORF44 and 45 with their *terL* homologs and needs to be verified by further experiments.

### 3.8. Integrase and Lysis

vB_MoxS-R1 ORF1 encodes an integrase, which is crucial for integrating the viral genome into the host genome. On the basis of their catalysis features, phage integrases are divided into tyrosine and serine types. High similarities of ORF1 with the tyrosine-type integrase homologs in the NR and Conserved Domain databases showed that the integrase of vB_MoxS-R1 belongs to the tyrosine type, as do those of vB_Mox-S1 and the *Microbacterium* prophage Min1. However, the integrases of *Microbacterium* EH-cluster phages are serine types. The tyrosine-type integrase, the most common integrase type among prokaryotes [128], cleaves DNA substrates using a series of cross-cutting processes in which proteins are covalently attached to DNA by catalytic tyrosine residues at the carboxyl termini [129,130]. Although vB_MoxS-R1 is closely related to the EH-cluster phages in the phylogenies constructed using the major capsid protein and terminase large subunit (Figure 7), they encode different types of integrases to enter the host chromosome.

To escape from the hosts, most phages use endolysins and holin to penetrate the cell membrane and cell wall within a certain period [131]. Lysins lyse the peptidoglycan structure of the host cell wall, whereas holin forms a membrane lesion and allows the lysins to attack the murein [129,130,131,132,133]. ORF64 in the vB_MoxS-R1 genome was predicted to encode a lysin A protein (Figure 3, Table S2), which is present in most *Microbacterium* phages. This suggests that vB_MoxS-R1 employs a host-lysing method similar to those of most *Microbacterium* phages.

## 4. Conclusions

In this study, a prophage vB_MoxS-R1 induced from a heterotrophic bacterium, *Microbacterium oxydans* R1, was characterized. vB_MoxS-R1 represents a novel lineage of siphovirus with a high replication capability. Six TR genes of different types and auxiliary metabolic genes predicted in the vB_MoxS-R1 genome indicate the potential role of this prophage in regulating host metabolism and increasing host fitness. The presence of four predicted endonuclease genes involved in DNA recombination or gene conversion in the genome suggests that vB_MoxS-R1 has the potential to mediate the genetic exchange and thus influence host evolution. The unique host-related genes found in the *Microbacterium* prophage indicate the potential viral influences on their hosts. It would be interesting to determine the actual impact of prophages on host functions by comparing the behaviors of hosts with and without prophages.

## Figures and Tables

**Table 1:** vB_MoxS-R1 infectivity analysis.

Tested Strains	Bacteria Taxa	Infection ^a^
R1	Actinobacteria; Micrococcales; *Microbacterium oxydans*	√ (lysogenic)
CBW1101-8	Actinobacteria; Micrococcales; *Microbacterium oxydans*	√ (lysogenic)
CBW1101-9	Actinobacteria; Micrococcales; *Microbacterium oxydans*	√ (lysogenic)
CBW1107-2	Alphaproteobacteria; Rhizobiales; *Mesorhizobium sediminum*	**×**
CBW1107-5	Alphaproteobacteria; Rhizobiales; *Nitratireductor aquimarinus*	**×**
CBW1107-11	Alphaproteobacteria; Sphingomonadales; *Sphingomonas xenophagum*	**×**
CBW1107-12	Flavobacteria; Flavobacteriales; *Muricauda alvinocaridis*	**×**
CBW1107-13	Flavobacteria; Flavobacteriales; *Arenibacter troitsensis*	**×**
CBW1107-3	Gammaproteobacteria; Alteromonadales; *Marinobacter salsuginis*	**×**
CBW1107-7	Gammaproteobacteria; Alteromonadales; *Marinobacter salsuginis*	**×**
CBW1107-4	Gammaproteobacteria; Oceanospirillales; *Halomonas hydrothermalis*	**×**
CBW1107-6	Gammaproteobacteria; Oceanospirillales; *Halomonas venusta*	**×**
CBW1107-10	Gammaproteobacteria; Oceanospirillales; *Halomonas piezotolerans*	**×**

^a^ **×**, no infection; √ infection.

**Table 2:** Homologous ORFs between vB_MoxS-R1 and vB_Mox-S1 and their network-related phages.

Phage	Family	Genus	ORF Homolog No.	ORF aa Identity (%)	Homologous ORF in vB_MoxS-R1
vB_Mox-S1	-	-	24	26.4–98.8	see in Figure 3
*Microbacterium* phage Nike	Siphoviridae	*Squashvirus*	4	33.6–54.9	ORF32, ORF38, ORF63, ORF68
*Microbacterium* phage Squash	Siphoviridae	*Squashvirus*	4	33.6–54.9	ORF32, ORF38, ORF63, ORF68
*Microbacterium* phage IAmGroot	Siphoviridae	unknown	4	21.8–32.7	ORF41, ORF45, ORF49, ORF58
*Microbacterium* phage GardenState	Siphoviridae	unknown	4	21.8–32.7	ORF41, ORF45, ORF49, ORF58
*Microbacterium* phage Percival	Siphoviridae	unknown	3	29.6–32.4	ORF45, ORF49, ORF58
*Microbacterium* phage Floof	Siphoviridae	unknown	3	29.3–32.6	ORF45, ORF49, ORF58
*Microbacterium* phage Zeta1847	Siphoviridae	*Zetavirus*	2	32.1–32.4	ORF45, ORF49
*Mycobacterium* phage Bernal13	Siphoviridae	*Bernalvirus*	4	24.9–43.4	ORF1, ORF20, ORF45, ORF58
*Mycobacterium* phage Mendokysei	Siphoviridae	*Bernalvirus*	3	25.3–31.6	ORF1, ORF20, ORF45

**Table 3:** Transcriptional regulators predicted in the vB_MoxS-R1 genome.

ORF No.	Family	Regulated Cellular Process	Reference
3	Unknown	Unidentified	-
5	ArsR family	Arsenical resistance	[105,106,107,108,109,110,111]
22	XRE family	Stress response and virulence	[114,115,116]
24	ASC-1-like subfamily	Regulation on transcription coactivation and RNA-processing	[117,118]
28	Unknown	Unidentified	-
73	XRE family	Stress response and virulence	[114,115,116]

## Data Availability

The complete genome sequence of prophage vB_MoxS-R1 was submitted to the GenBank database under accession number MW073100.

## Supplementary Materials

The following supporting information can be downloaded at: https://www.mdpi.com/article/10.3390/v14040731/s1, Table S1: Increased vB_MoxS-R1 *mcp* copies in phage-treated *Microbacterium oxydans* R1, CBW1101-8 and CBW1101-9 cells; Table S2: Annotation of the predicted vB_MoxS-R1 ORFs with homologs in the GenBank non-redundant database; Figure S1: Genome sequence comparison of vB_MoxS-R1 and *Microbacterium* sp. UCD-TDU using Circoletto (http://tools.bat.infspire.org/circoletto/ (accessed on 18 March 2020)); Figure S2: Protein-sharing viral network of vB_MoxS-R1, vB_Mox-S1 and ViralRefSeq database viruses with a pairing-similarity score > 1. Each node represents the genome of a phage. Edges represent interaction between pairs of viruses. The nodes of vB_MoxS-R1, vB_Mox-S1 and their related phages are colored in different colors; Figure S3: Phylogenetic trees of the major capsid proteins (a) and terminase large subunit (b) of vB_MoxS-R1 and other known phages. The bootstrap values (maximum-likelihood/neighbor-joining) are shown near each node. Number of bootstrap replicates = 1000. The phage clusters used in the major capsid protein phylogenetic analyses referenced the siphophage clusters used in the phage gene phylogenetic analyses by Zhan et al. (2016) and Yang et al. (2017) [134,135]. The phage clusters used in the terminase large-subunit phylogenetic analyses referenced the phage clusters employed in the phage terminase large-subunit phylogenetic analyses by Huang et al. (2012) [136]; Figure S4: Maximum-likelihood tree constructed using bacterial 16S rRNA sequences. The bootstrap values (maximum-likelihood/neighbor-joining) are shown near each node. Number of bootstrap replicates = 1000. The *Microbacterium* hosts of the EH-cluster phages and temperate-phage Min 1 include *Microbacterium foliorum*, *Microbacterium paraoxydans*, *Microbacterium aerolatum* and *Microbacterium neimengense*. The 16S rRNA sequences of *Microbacterium paraoxydans* NWU1 and *Microbacterium aerolatum* NRRL B-24228 were not available; therefore, type–strain sequences of the same species were used in the analyses. *Arthrobacter protophormiae* was used as the outgroup. The host of the vB_MoxS-R1 is indicated in blue. Host bacterial strains of the inducible or predicted prophages are indicated in bold, and type strains of unavailable species are indicated in brown; Figure S5: The predicted −1 frameshift in the vB_MoxS-R1 terminase large-subunit gene.
