# Supplementary material for: An Inducible Microbacterium Prophage vB_MoxS-R1 Represents a Novel Lineage of Siphovirus
Source: Viruses. 2022 Mar 31;14(4):731. doi: 10.3390/v14040731 (PMC9030533; doi:10.3390/v14040731)
Supplement: Supplementary file 1 [file viruses-14-00731-s001.zip › viruses-1588889-supplementary.pdf]

Supplementary Material

Table S1. Increased vB_MoxS-R1 *mcp* copies in phage-treated *Microbacterium oxydans* R1, CBW1101-8 and CBW1101-9 cells.

Tested strain	Time (h)	Control (copies cell^{-1})	Phage treated (copies cell^{-1})	Increased ratio (%)
Microbacterium sp. R1	0.5	1.05	1.38	31
	2	2.94	4.01	36
	4	1.95	2.62	34
Microbacterium sp. CBW1101-8	0.5	0.70	0.75	7
	2	3.29	4.13	26
	4	2.56	2.83	11
Microbacterium sp. CBW1101-9	0.5	0.42	0.53	26
	2	0.42	0.61	45
	4	0.36	0.54	50

Table S2. Annotation of the predicted vB_MoxS-R1 ORFs with homologs in the GenBank non-redundant database.

Gene	Strand	Left	Right	Length (aa)	Significant hits in NR[a]	Putative funtion[b]	E-value	aa identity	Conserved domain (e-value)[c]
1	+	170	1447	425	hypothetical protein D514_0102395 [*Microbacterium* sp. UCD-TDU]	integrase	0	95.06%	cd01189 (3.87E-12)
					tyrosine-type recombinase/integrase [*Microbacterium* sp. UCD-TDU]		0	95.05%	
					integrase [*Microbacterium oleivorans*]		2 E-120	58.47%	
2	-	1500	1952	150	ImmA/IrrE family metallo-endopeptidase [*Microbacterium* sp. 4-7]	metallo-endopeptidase	4 E-71	73.10%	cl01076 (4.40E-04)
					ImmA/IrrE family metallo-endopeptidase [*Mycetocola reblochoni*]		2 E-60	64.34%	
3	-	1949	2521	190	hypothetical protein [*Microbacterium* sp. UCD-TDU]	transcriptional regulator	3 E-101	82.61%	
					helix-turn-helix transcriptional regulator [*Microbacterium* sp. K24]		3 E-35	46.84%	
5	+	2883	3686	267	helix-turn-helix domain-containing protein [*Curtobacterium* sp. MCBD17_021]	transcriptional regulator	7 E-55	43.82%	
					hypothetical protein C7321_0396 [*Microbacterium trichothecenolyticum*]		1 E-40	36.86%	
					ArsR family transcriptional regulator [*Mycobacterium asiaticum*]		1 E-17	44.35%	
7	+	4546	4767	73	helix-turn-helix domain-containing protein [*Microbacterium* sp. UCD-TDU]	DNA binding	1 E-38	87.67%	pfam12728 (2.25E-05)
					DNA-binding protein [*Microbacterium* sp. HSID17254]		2 E-27	75.38%	
13	+	6173	6955	260	HNH endonuclease [*Agromyces albus*]	endonuclease	3 E-30	32.97%	pfam01844 (1.97E-07)
					HNH endonuclease [*Microbacterium* sp. CGR2]		1 E-12	52.31%	
20	+	8928	9821	297	hypothetical protein [*Microbacterium* sp. K5D]	exonuclease VIII	0	94.61%	cl00641 (1.03E-20)
					PDDEXK-like uncharacterized protein DUF3799 [*Microbacterium lindanitolerans*]		1 E-64	42.07%	
					recE [*Curtobacterium* sp. MCSS17_006]		2 E-60	40.68%	
22	+	9957	10559	200	MULTISPECIES: helix-turn-helix transcriptional regulator [unclassified *Microbacterium*]	transcriptional regulator	3 E-131	92.00%	cd00093 (7.62E-06)
23	+	10646	10834	62	HNH endonuclease [*Microbacterium oxydans*]	endonuclease	1 E-38	98.39%	cl00083 (5.14E-05)
24	+	10837	11331	164	ASCH domain-containing protein [*Mycetocola reblochoni*]	transcription factor	1 E-40	56.36%	cd06554 (8.44E-22)
					hypothetical protein [*Microbacterium* sp. 4NA327F11]		1 E-38	52.76%	
26	+	11558	12112	184	MULTISPECIES: hypothetical protein [unclassified *Microbacterium*]	DNA binding	1 E-132	97.83%	
					helix-turn-helix DNA binding domain protein [*Gordonia* phage Fairfaxidum]		4 E-47	45.61%	
					helix-turn-helix DNA binding domain protein [*Rhodococcus* phage Whack]		4 E-38	40.80%	
27	+	12285	12587	100	helix-turn-helix domain-containing protein [*Microbacterium* sp. Leaf320]	DNA binding	5 E-46	71.88%	cl21459 (6.70E-07)

Table S2. Continued.

Gene	Strand	Left	Right	Length (aa)	Significant hits in NR[a]	Putative funtion[b]	E-value	aa identity	Conserved domain (e-value)[c]
28	+	12584	13096	170	hypothetical protein [*Microbacterium* sp. H6]	transcription factor	3 E-85	88.95%	
					response regulator transcription factor [*Microbacterium* sp. No. 7]		2 E-12	39.24%	
					Helix-turn-helix domain of resolvase [*Microbacterium paraoxydans*]		2 E-11	39.04%	
29	+	13093	13833	246	DNA (cytosine-5-)-methyltransferase [*Agromyces lapidis*]	DNA methyltransferase	8 E-102	61.57%	cl30459 (1.08E-45)
					methyltransferase [*Mycobacterium* phage Zerg]		4 E-93	60.25%	
tRNA	+	16814	16887		tRNA-Ala				
38	+	17288	17542	84	hypothetical protein E6R04_05360 [*Spirochaetes bacterium*]	membrane protein	1 E-30	76.39%	
					membrane protein [*Microbacterium* phage Squash]		1 E-20	54.88%	
41	+	17866	18171	101	hypothetical protein FB466_0640 [*Klugiella xanthotipulae*]	endonuclease	9 E-29	68.69%	
					HNH endonuclease [*bacterium*]		1 E-06	37.68%	
43	+	18884	19480	198	HNH endonuclease [*Rhodococcus fascians*]	endonuclease	3 E-51	50.87%	pfam01844 (6.28E-08)
					HNH endonuclease [*Microbacterium* sp. CH12i]		4 E-18	44.68%	
44	+	19556	19726	56	MULTISPECIES: terminase [unclassified *Microbacterium*]	terminase	1 E-27	91.07%	
45	+	19713	21167	484	MULTISPECIES: terminase [unclassified *Microbacterium*]	terminase	0	99.38%	
					terminase [*Microbacterium telephonicum*]		0	72.34%	
					Phage terminase-like protein%2C large subunit [*Chlamydia trachomatis*]		0	61.24%	
46	+	21179	22495	438	MULTISPECIES: phage portal protein [unclassified *Microbacterium*]	portal protein	0	97.49%	cl19531 (2.97E-10)
47	+	22479	23288	269	MULTISPECIES: hypothetical protein [unclassified *Microbacterium*]	capsid maturation protease	0	96.28%	
					capsid maturation protease [*Mycobacterium* phage Llama]		1 E-63	42.69%	
49	+	23937	25148	403	MULTISPECIES: hypothetical protein [unclassified *Microbacterium*]	major capsid protein	0	98.01%	cl22542 (2.23E-08)
					major capsid protein [*Mycobacterium* phage Bromden]		3 E-48	35.00%	
53	+	26318	26713	131	hypothetical protein BGN97_00240 [*Microbacterium* sp. 69-10]	head-to-tail connector complex	2 E-18	40.46%	
					head-to-tail connector complex protein [*Streptomyces* phage Rowa]		2 E-06	29.23%	
58	+	28682	31627	981	tape measure protein [*Microbacterium* sp. ZXX196]	tail tape measure	0	59.20%	cl34696 (2.73E-07)
59	+	31624	33081	485	hypothetical protein NS234_04900 [*Microbacterium oxydans*]	tail protein	0	99.79%	
					minor tail protein [*Microbacterium* phage VitulaEligans]		5 E-32	39.38%	
63	+	35727	36509	260	hypothetical protein [*Microbacterium* sp. H6]	lysin A	3 E-91	60.00%	
					lysin A [*Microbacterium* phage Chepli]		9 E-76	50.80%	

Table S2. Continued.

Gene	Strand	Left	Right	Length (aa)	Significant hits in NR[a]	Putative funtion[b]	E-value	aa identity	Conserved domain (e-value)[c]
65	+	36877	37512	211	hypothetical protein [*Microbacterium* sp. K24]	collagen	5 E-52	53.88%	pfam01391 (1.20E-04)
					collagen-like protein [*Microbacterium* sp. PF5]		6 E-51	56.54%	
66	+	37509	37853	114	hypothetical protein [*Microbacterium* sp. H6]	metallopeptidase	1 E-69	98.25%	
					M15 family metallopeptidase [*Nocardioides* sp. W3-2-3]		1 E-05	47.50%	
68	+	38244	40118	624	MULTISPECIES: hypothetical protein [unclassified *Microbacterium*]	glycerophosphodiester phosphodiesterase	0	95.35%	cl14615 (4.68E-15)
					glycerophosphodiester phosphodiesterase [*Microbacterium* phage ValentiniPuff]		0	58.15%	
					phosphoesterase [*Microbacterium* phage Triscuit]		1 E-58	48.41%	
71	-	40725	41066	113	hypothetical protein [*Kocuria palustris*]	membrane protein	1 E-13	0.4432	
					Predicted membrane protein [*Chlamydia trachomatis*]		1 E-8	0.3158	
73	-	41395	41592	65	XRE family transcriptional regulator [*Microbacterium liquefaciens*]	transcriptional regulator	5 E-29	81.25%	
tRNA	+	42493	42559		Pseudo tRNA-Thr				

[a] The displayed top homolog and additional homologs for each different organism type are based on the BLASTP against the non-redundant database. [b] Putative functions were predicted on the basis of functions of homologs identified from the non-redundant and Conserved Domain Databases. [c] The best hit of each ORF in the Conserved Domain Database and its e-value.

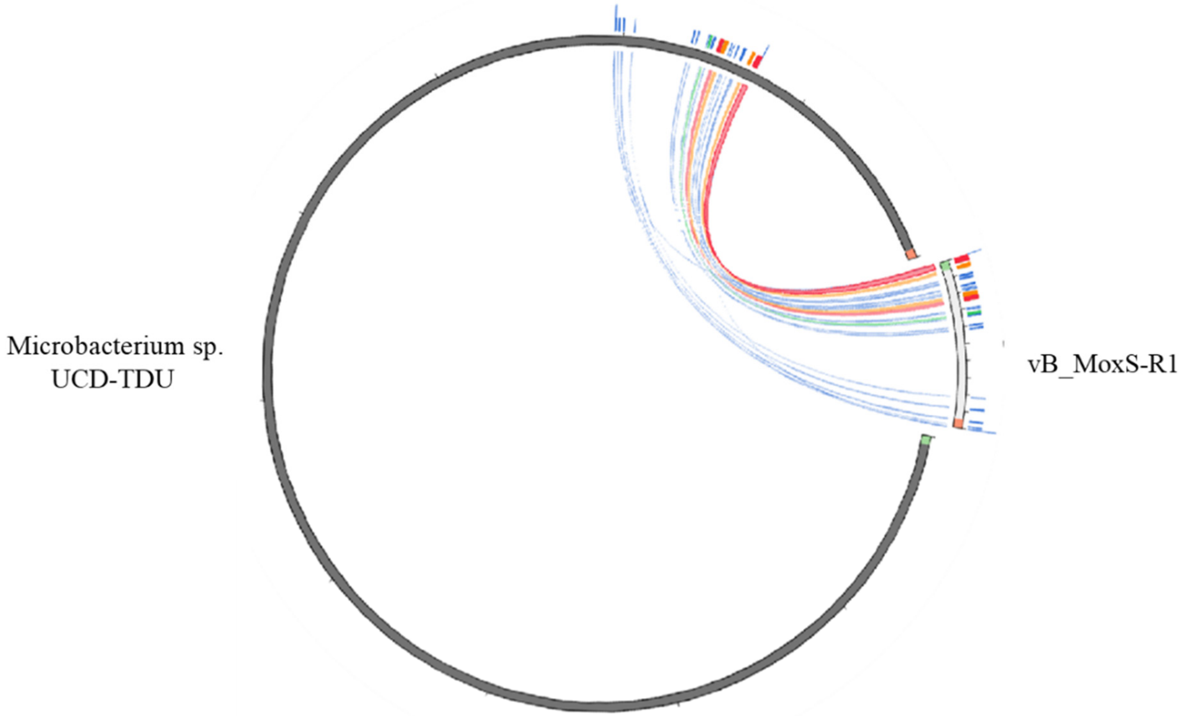

Figure S1. Genome sequence comparison of vB_MoxS-R1 and *Microbacterium* sp. UCD-TDU using Circoletto (http://tools.bat.infspire.org/circoletto/ (accessed on 12 March 2020)).

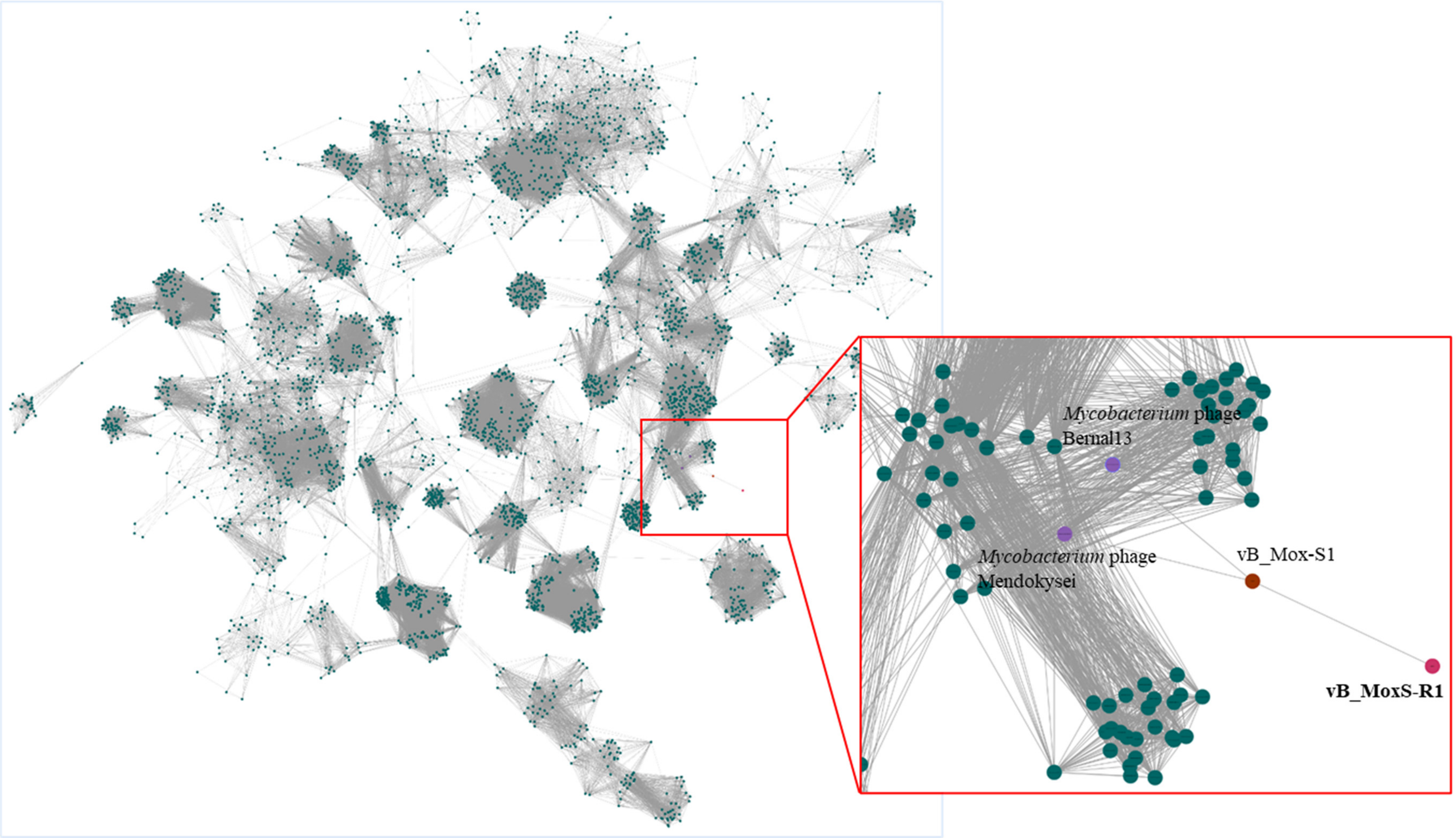

Figure S2. Protein-sharing viral network of vB_MoxS-R1, vB_Mox-S1 and ViralRefSeq database viruses with a pairing-similarity score > 1. Each node represents the genome of a phage. Edges represent interaction between pairs of viruses. The nodes of vB_MoxS-R1, vB_Mox-S1 and their related phages are colored in different colors.

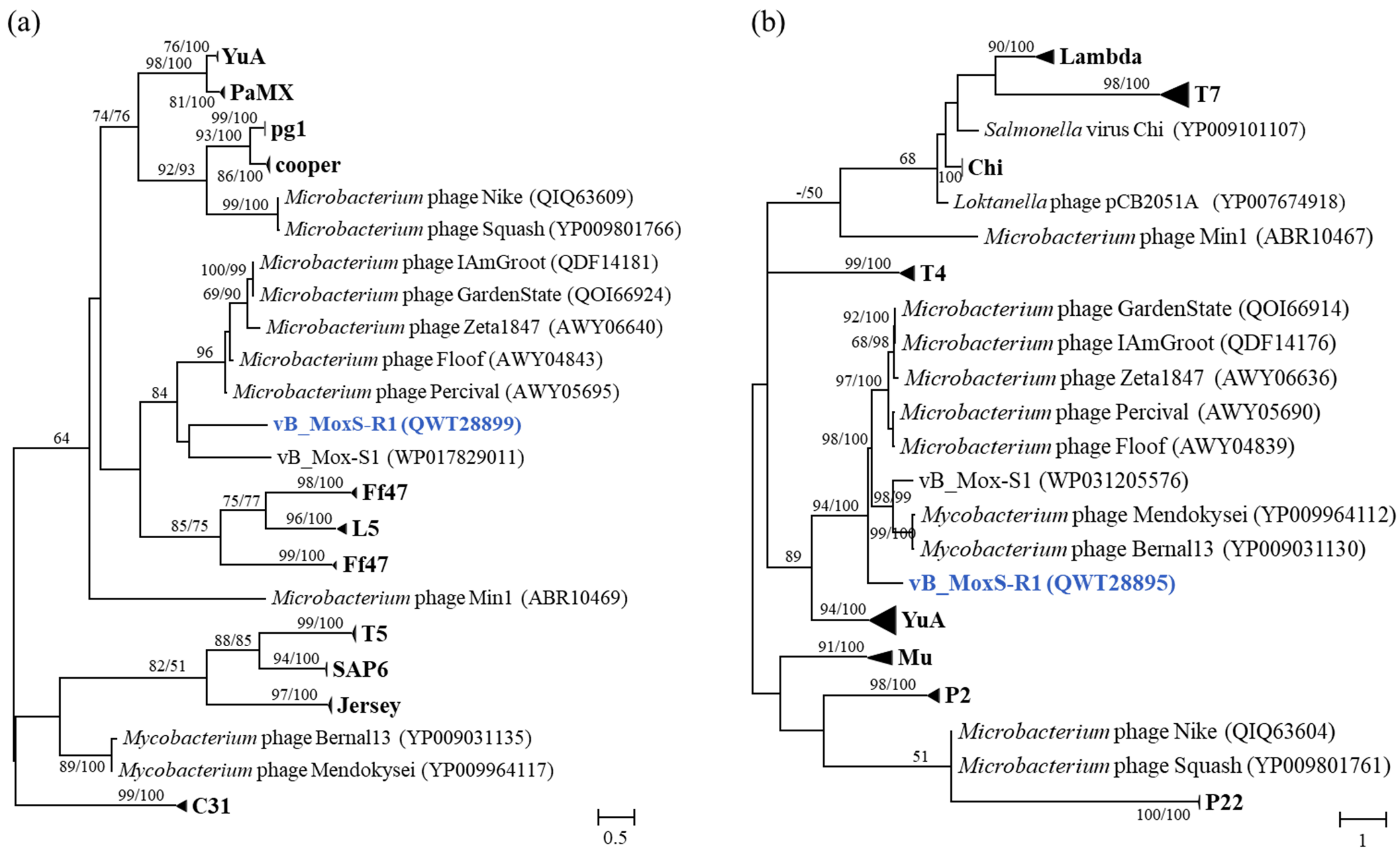

Figure S3. Phylogenetic trees of the major capsid protein (**a**) and terminase large subunit (**b**) of vB_MoxS-R1 and other known phages. The bootstrap values of (Maximum-likelihood/Neighbor-joining) are shown near each node. Number of bootstrap replicates = 1000. The phage clusters used in the major capsid protein phylogenetic analyses referenced the siphophage clusters used in the phage gene phylogenetic analyses in Zhan et al. (2016) [134] and Yang et al. (2017) [135]. The phage clusters used in the terminase large subunit phylogenetic analyses referenced the phage clusters employed in the phage terminase large subunit phylogenetic analyses in Huang et al. (2012) [136].

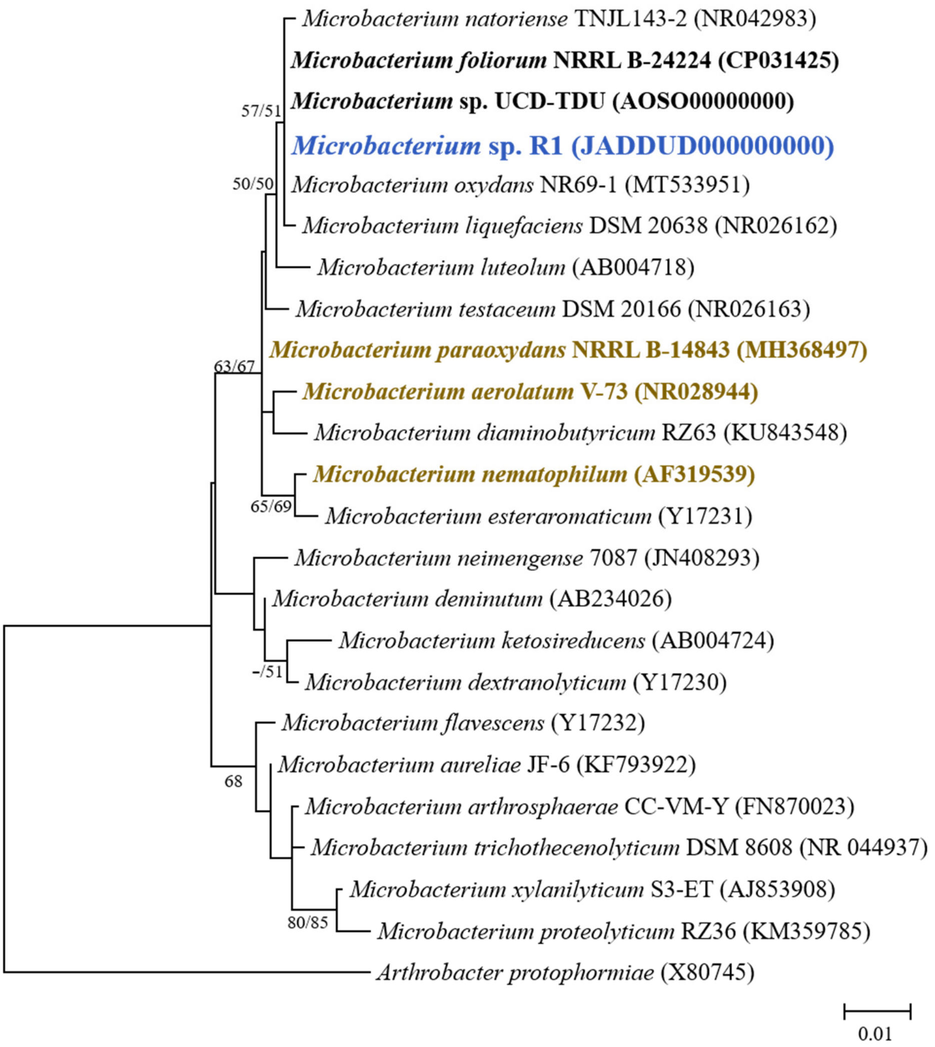

Figure S4. Maximum-likelihood tree constructed using bacterial 16S rRNA sequences. The bootstrap values (Maximum-likelihood/Neighbor-joining) are shown near each node. Number of bootstrap replicates = 1000. The *Microbacterium* hosts of the EH-cluster phages and temperate phage Min 1 include *Microbacterium foliorum, Microbacterium paraoxydans*, *Microbacterium aerolatum* and *Microbacterium neimengense.* The 16S rRNA sequences of *Microbacterium paraoxydans* NWU1 and *Microbacterium aerolatum* NRRL B-24228 were not available; therefore, type-strain sequences of the same species were used in the analyses. *Arthrobacter protophormiae* was used as the outgroup. The host of the vB_MoxS-R1 is indicated in blue. Host bacterial strains of the inducible or predicted prophages are indicated in bold, and type strains of unavailable species are indicated in brown.

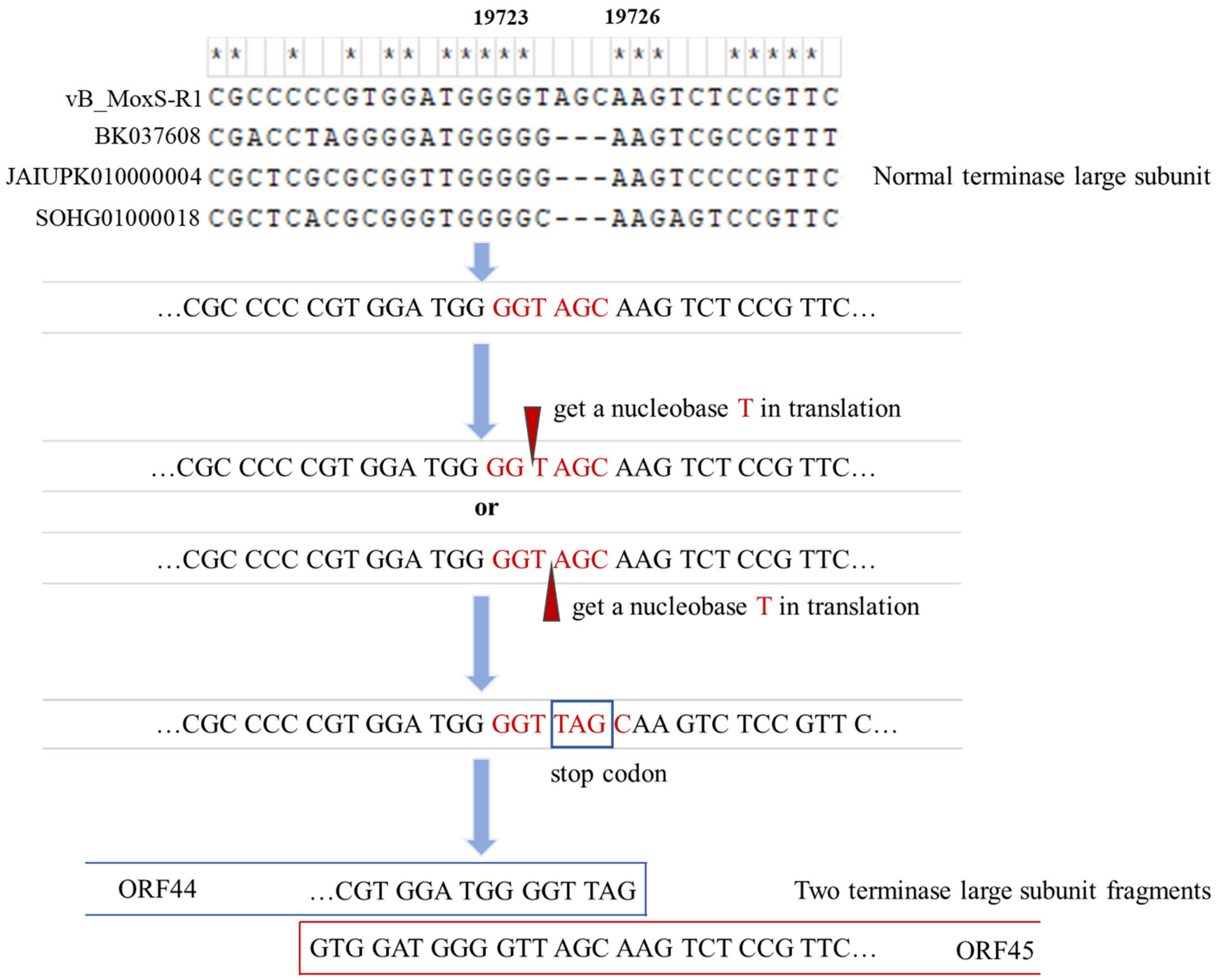

Figure S5. The predicted −1 frameshift in the vB_MoxS-R1 terminase large-subunit gene.
